# A Multifaceted Quality Improvement Intervention to Improve Watchful Waiting in Acute Otitis Media Management

**DOI:** 10.1097/pq9.0000000000000177

**Published:** 2019-05-23

**Authors:** Di Sun, Vanessa Rivas-Lopez, Danica B. Liberman

**Affiliations:** From the * Department of Pediatrics, Children’s Hospital Los Angeles, Los Angeles, Calif.; † Division of Emergency and Transport Medicine, Children’s Hospital Los Angeles, Los Angeles, Calif.; ‡ Department of Pediatrics, Keck School of Medicine of the University of Southern California, Los Angeles, Calif.

## Abstract

**Introduction:**

Despite the American Academy of Pediatrics (AAP) guidelines for acute otitis media (AOM) describing a watchful waiting (WW) approach in qualifying patients, immediate antibiotics are consistently overutilized. The study team developed a multifaceted quality improvement intervention that educated providers and families about WW and included a behavioral component to modify physician prescribing patterns.

**Methods:**

We used data from a prior study of 250 patients 18 years old and younger with AOM in a tertiary care children’s hospital emergency department (ED) to characterize baseline AOM management before interventions. In this study, interventions took place from September to December 2016. Following the interventions, 65 patients were randomly selected, which would allow for the detection of a 20% increase in adherence to AAP guidelines for management of AOM.

**Results:**

In the preintervention cohort of 250 patients, 247 had documented AOM. Two hundred thirty-one (93.5%) received immediate antibiotics, 7 (2.8%) underwent WW, and 9 (3.6%) were sent home without antibiotics. Overall management agreed with AAP guidelines at a rate of 44.1%. In the postintervention cohort of 65 patients, 63 met age and diagnostic criteria for AOM; 56 (88.9%) patients received immediate antibiotics; and 7 (11.1%) underwent WW. Postintervention, which the ED management of AOM agreed with AAP guidelines 60.3% of the time, was significantly increased from preintervention adherence (*P* = 0.02).

**Conclusions:**

A multipronged quality improvement intervention for AOM management in a single pediatric ED significantly improved adherence to AAP guidelines by increasing WW and reducing immediate antibiotic prescriptions.

## Abstract 2

Supplemental Digital Content is available in the text.

## INTRODUCTION

Acute otitis media (AOM) is a leading diagnosis for antibiotic use in children in the United States, and in 2010–2011 resulted in an estimated 154 prescriptions per 1,000 population.^1^ Physicians prescribe antibiotics for AOM 95% of the time despite evidence suggesting that most children will improve with supportive care alone.^2–5^ A 2015 Cochrane Review also suggested that when compared with placebo, antibiotics did not avert long-term abnormal tympanometry findings or decrease the incidence of serious complications of AOM.^2^

These studies formed the basis of the 2013 American Academy of Pediatrics (AAP) guidelines describing a watchful waiting (WW) approach for managing AOM in qualifying patients over the age of 6 months with no other comorbidities. According to these guidelines, children 2 years and older without severe symptoms— defined as moderate or severe otalgia, otalgia ≥ 48 hours, or temperature ≥ 39°C—can undergo a 48–72 hour WW period to allow for spontaneous symptom resolution before antibiotic initiation. Children who are 6–23 months with bilateral otitis media or any children with severe symptoms should undergo immediate antibiotic therapy. The AAP guidelines do not recommend WW if the family requests immediate antibiotic therapy or if follow-up is equivocal.^5^

Although the AAP has recommended the WW option since the original 2004 AOM guidelines, a survey of primary care physicians before and after these guidelines indicated little change in clinical practice.^6^ Parental reluctance (83.5%) and the additional cost and time needed to manage patients with persistent symptoms (30.9%) were the main barriers to WW implementation.^6^ A recent study evaluating the cost-effectiveness of WW for AOM suggests that when a healthcare provider prescribes a safety-net antibiotic prescription to the family—a prescription to be filled only if symptoms are not improving over the WW period, WW is highly cost-effective compared to an immediate antibiotic prescription from a societal perspective.^7^ Moreover, additional studies have found no difference in parental satisfaction between those who underwent WW versus those receiving an immediate antibiotic prescription.^8,9^

Despite this data, studies evaluating methods for changing physician management have suggested that traditional methods, such as pay-for-performance or electronic medical record (EMR) alerts, do not always generate desirable outcomes.^10–12^ In contrast, behavioral interventions, particularly those that have a social component, are effective in altering physician decision-making. In 2 studies by Meeker et al,^13,14^ a decrease in inappropriate antibiotic use for acute respiratory infections was achieved through using: (1) a signed commitment letter posted in examination rooms; 2) accountable justification in which physicians entered their reasoning for potentially questionable antibiotic prescriptions; and 3) comparison of individual physician antibiotic prescribing rates with those of “top-performing” peers in antibiotic stewardship.

**Fig. 1.**
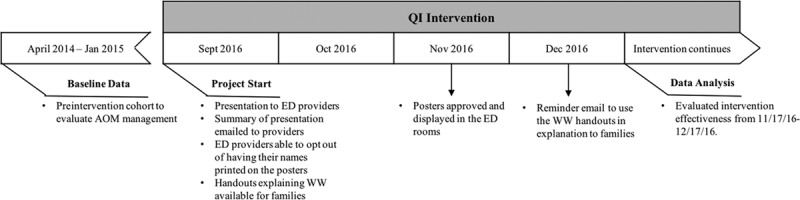
Study timeline.

To form the baseline data for this quality improvement (QI) project, the study team used data from a prior study^7^ that characterized AOM management in a pediatric emergency department (ED)—the site of most AOM diagnoses—and demonstrated that providers prescribed antibiotics for 93.5% of patients with AOM. We hypothesized that WW underutilization was due to unfamiliarity with WW implementation, time constraints in the ED setting, and providers’ perception of parental expectation for antibiotics. To address these barriers, we developed a multifaceted QI intervention that included education for providers and families and a behavioral component to modify physician prescribing patterns (Fig. 1). The study aimed to increase adherence to AAP guidelines for AOM management by 20% from 44.1% to 64.1%.

## METHODS

### Setting

This QI initiative to improve AOM management took place in the ED of a freestanding, tertiary care children’s hospital, which treats over 80,000 patients per year with roughly 4,500 AOM cases annually. Pediatric emergency medicine physicians, general pediatricians, and pediatric nurse practitioners staff this ED. Targeting ED providers would thus have a significant impact on reducing inappropriate antibiotic prescriptions for AOM and increasing WW. The Institutional Review Board approved this study (CHLA-18–0080).

### Preintervention Cohort

Data from a prior study in which 250 patients 18 years old and younger diagnosed with AOM in the ED at this institution from April 2014 to January 2015 were used as baseline data to characterize AOM management before any interventions.^7^ Study personnel identified these patients by *International Classification of Diseases, Ninth Revision* codes placed at discharge. We randomly selected 250 patients seen and discharged from the ED with AOM for further review. Study authors from the prior study reviewed the medical records for these 250 patients for age, symptom severity, and physical exam to determine if they met the criteria for WW based on AAP guidelines.

### Interventions

**Fig. 2.**
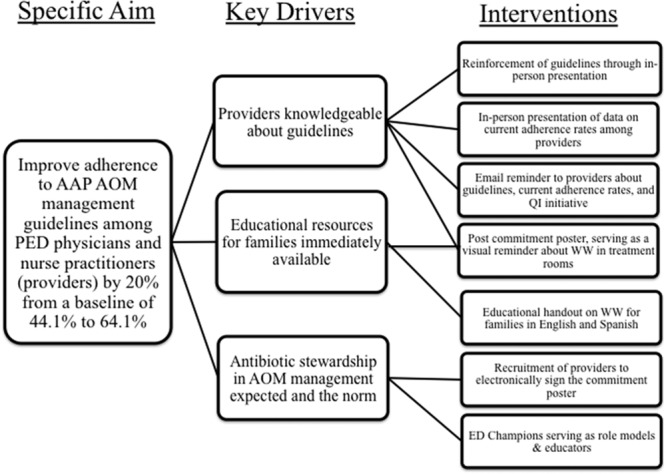
Key driver diagram. PED, pediatric emergency department.

Interventions were developed to target each hypothesized barrier to using WW (Fig. 2). All 46 providers (43 physicians and 3 nurse practitioners) working in the ED endorsed this QI initiative when it was introduced, and participated in some or all intervention elements. To familiarize ED physicians and nurse practitioners with WW implementation, we provided a 1-hour long presentation that explained the AAP AOM guidelines, reviewed this institution’s current antibiotic prescription rates, and developed a visual algorithm (Supplemental Digital Content 1, Figure 1: Algorithm for management of acute otitis media as suggested by the American Academy of Pediatrics guidelines. Available at http://links.lww.com/PQ9/A95) to help clinicians decide when WW was appropriate. The summarized presentation was provided to the ED division via email for clinicians to reference as needed, and so that those who were unable to attend would have an opportunity to receive this educational intervention. To address time constraints in the ED setting, we developed an easy-to-read handout on AOM for parents that explained the reasoning for WW (Supplemental Digital Content 2, Figure 2. Acute otitis media handout explaining watchful waiting for families. Available at http://links.lww.com/PQ9/A96). Finally, we added a behavioral intervention that was simultaneously designed to modify physician behavior and manage parental expectations through posting a commitment poster, electronically signed by all ED providers (Supplemental Digital Content 3, Figure 3. Acute otitis media watchful waiting flier posted in the exam rooms. Available at http://links.lww.com/PQ9/A97) in patient examination rooms and waiting areas that explained antibiotic stewardship for AOM management. Both the parent handout and commitment posters were written at a third-grade reading level according to hospital practice. They were formally edited and approved by the patient family education and resources department as well as the marketing communications division. Hospital-approved translation services translated the handout and poster into Spanish versions. All interventions were conducted over 4 months, from September to December 2016, with parent handouts readily available and posters displayed in the examination rooms on November 16, 2016. A single reminder email was sent to all ED providers in early December 2016 to encourage providers to employ the handouts and posters when counseling families (Fig. 1).

### Postintervention Cohort

Using Stata/MP 14 (StataCorp, College Station, Tex.), our sample size calculation was framed under the z-test for difference between proportions (at least 20% difference) with statistical power of at least 80% and alpha level of 0.05 (two-tailed to allow for the possibility of a difference in the opposite direction). The sample size calculation outputted 240 as the size of the first proportion and 60 as the size of the second. This 4:1 ratio of preintervention to postintervention sample size was a decision we made to balance rigor and feasibility. Given 250 available cases for the first proportion, approximately 63 cases were needed for the second proportion, and we rounded this number up to 65.

After completing the interventions, we randomly selected 65 patients from the entire population of those seen and discharged from the ED with an AOM diagnosis based on the *International Classification of Diseases, Tenth Revision* codes between November 17, 2016, and December 17, 2016. We analyzed the medical records of these 65 patients using the same standardized form used to collect data on the preintervention cohort. Specifically, we collected data on patient age, gender, clinical symptoms, pertinent physical exam findings, pain score based on FACES (Face, Leg, Activity, Cry, Consolability scales), and documented treatment plan (WW, no antibiotics prescribed, antibiotics given).^15,16^ If an antibiotic was prescribed, we assumed it was an immediate antibiotic prescription unless the ED provider documented WW. Similar to the method used in the preintervention cohort, we used patient demographics, symptom severity, and physical examination findings to determine if patients met criteria for WW based on AAP guidelines.

Given that this was a retrospective study in which study personnel could not enforce strict AOM diagnostic criteria, patients with documented AOM physical examination findings were assumed to have AOM. Two study authors (D.S. and V.R.-L.) independently abstracted the data and determined if patients met WW criteria. We resolved any questions or discrepancies through open discussion, and senior author (D.B.L.) adjudicated any further disagreement.

### Analysis

Preintervention and postintervention percentages of patients who received immediate antibiotics, recommended to undergo WW, or were discharged without antibiotics, were calculated from the data. To determine adherence to AAP guidelines, we evaluated whether patients met criteria for WW (based on age, symptom severity, and physical examination as outlined in the AAP guidelines) with their actual management. The proportion in which management agreed with AAP guidelines was calculated in both the preintervention and postintervention cohorts. Differences in medians and proportions between the preintervention and postintervention cohorts were performed using the Mann–Whitney test and the z-test, respectively. We performed our calculations in Stata/MP 14 (StataCorp).

## RESULTS

### Patient Characteristics

A prior study by Sun et al^7^ was used as the preintervention AOM cohort and included 250 patients with the exclusion of 3 patients given physical examinations inconsistent with AOM. The original analysis included 247 patients with the median age being 3 years (range: 6 months–18 y), with 135 males (54.7%). The majority (210 patients, 85%) had no comorbidities; however, 13 children (5.3%) had clinical factors (persistent fever, prior antibiotic initiation, concurrent urinary tract infection, or conjunctivitis) and 24 (9.7%) had comorbidities (asthma, recurrent AOM, and prematurity) that could impact clinical management.

In the postintervention cohort, of the 65 randomly selected patients, one patient did not have documented AOM, and one patient was younger than 6 months old, leaving 63 patients for final analysis. The median age was 2 years (range: 6 months–16 y) with 38 males (60%). In this population, 54 patients (86%) were previously healthy, whereas 4 patients (6.3%) had comorbidities (prematurity, recurrent AOM, and genetic syndromes) and 5 (7.9%) had clinical factors (concurrent pneumonia, prior antibiotic initiation, and conjunctivitis) that could impact clinical management (Table 1). Patient demographics were not significantly different between the preintervention and postintervention cohorts.

**Table 1.**
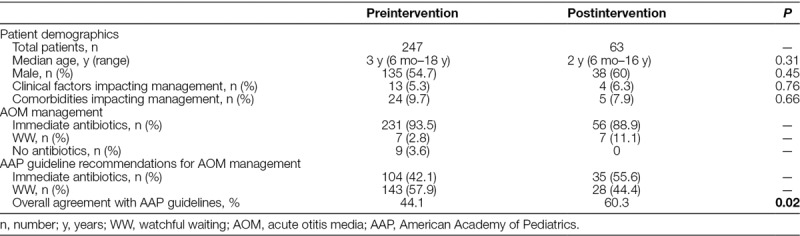
Comparison of Patient Demographics, Actual AOM Management, and Expected AOM Management When AAP Guidelines Are Applied to the Cohorts in the Preintervention and Postintervention Populations

### Outcome Measure

In the preintervention cohort, 231 (93.5%) patients received immediate antibiotics, 7 (2.8%) underwent WW, whereas 9 (3.6%) received neither antibiotics nor WW education. When AAP guidelines were used to determine eligibility for WW, 143 (57.9%) patients qualified for this option, whereas 104 (42.1%) patients met the criteria for immediate antibiotics (Table 1). When patients met criteria for immediate antibiotics, the ED gave antibiotics 100% of the time, but when patients qualified for WW, providers recommended WW only 4.9% of the time. Overall management agreed with AAP guidelines at a rate of 44.1%.

**Fig. 3.**
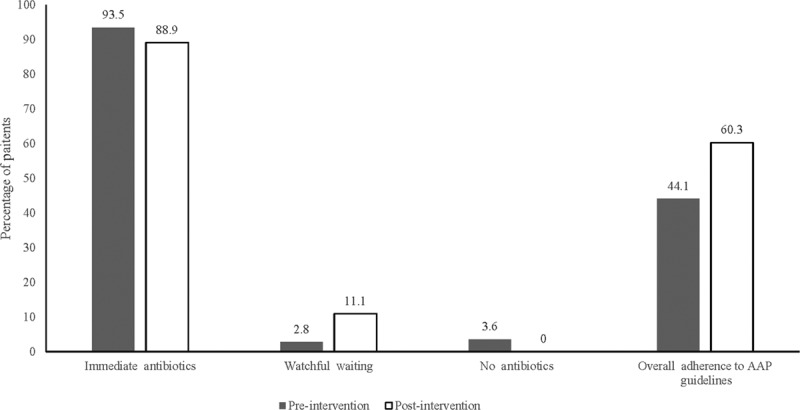
AOM management preintervention and postintervention.

In the postintervention cohort of 63 patients, 56 (88.9%) patients received immediate antibiotics, and the remaining 7 (11.1%) patients underwent WW. Using the AAP guidelines, we retrospectively deemed 28 (44.4%) patients eligible to undergo WW, whereas 35 (55.6%) patients qualified for immediate antibiotics (Table 1). When patients met AAP criteria for immediate antibiotics, they received antibiotics 94.3% of the time. When patients qualified for WW, they underwent WW 17.9% of the time. Postintervention, the ED management of AOM agreed with AAP guidelines 60.3% of the time (Fig. 3).

This study’s intervention increased adherence to AAP guidelines by 16.2% (*P* = 0.02, 95% CI, 2.6%–29.8%). Although this did not reach our targeted aim of a 20% increase in adherence to guidelines, the improvement shows that our interventions were successful in changing physician behavior in the short term.

## DISCUSSION

In this QI intervention, the aim was to improve provider adherence to AOM management guidelines by targeting factors motivating providers to overprescribe antibiotics for AOM. In a systematic review evaluating barriers to guideline adherence, physicians said they were unaware or unfamiliar with the guideline in a median of 54.5% and 56.5% of cases, respectively.^17^ This study identified a lack of self-efficacy in a median of 13% of surveyed physicians and inertia of prior practice in 42%. Many physicians also disagreed with the guidelines due to the perceived cost or risk to the patient, lack of application to their patient population, reduction in autonomy, management oversimplification, and guideline authors lack of credibility. External barriers included the inconvenience of the guideline and environmental factors (time and nursing staff support).^17^

This study’s interventions addressed many barriers; we familiarized providers with AAP guidelines for AOM management with a presentation that maximized self-efficacy, emphasized the benefit for families, and reinforced the effectiveness of the guidelines overall. We addressed external factors such as time constraints by providing handouts to expedite WW explanation to families. We addressed the parental expectation for antibiotics for AOM through a posted commitment poster for antibiotic stewardship. This signed poster also served as a behavioral intervention that visually reminded providers of their dedication to practice evidence-based care for AOM management. This intervention’s social component appealed to providers’ internal desire to give the best possible care for their patient and thus avoid any cognitive dissonance elicited through inappropriate antibiotic use. The poster’s public display also reminded providers of their commitment and the possibility of peer disapproval if they reneged on their responsibility to patients. In the month following the intervention, immediate antibiotic prescription rates were reduced from preintervention levels, and adherence to AAP guidelines improved by 16.2%. This intervention demonstrated that simple behavioral interventions could affect some change in physician behavior and thus reduce healthcare costs.

The interventions in this study were modestly effective in modifying short-term behavior. One reason the interventions may not have achieved their targeted goal could be that the initial educational presentation on the AAP guidelines did not capture all providers in the ED. Although the study team sent an email summarizing the presentation to all ED providers, there is no way to ensure that providers took the time to review the email. In the future, additional interventions could include offering a few interactive presentations at various times so that more staff would be able to attend, or making attendance at the presentations and reviewing related emails mandatory through tracking participation.

Another method for improving adherence to AAP guidelines would be to have nursing staff provide eligible patients the WW handout and begin the conversation regarding WW with families. Although this WW handout streamlines the process of explaining WW, this is still a lengthy task in the rapid-paced ED setting. Having nursing staff explain WW first, with physicians and nurse practitioners available to answer any additional questions afterward, could help expedite the educational process.

Finally, the behavioral intervention may not have been sufficient to produce the targeted goal. The signed commitment poster was an adaptation of the poster used by Meeker et al^14^ to influence physician behavior. In their randomized controlled trial, they demonstrated a 19.7 absolute percentage decrease in inappropriate antibiotic prescription rates between the control and intervention groups. This improvement is still less than the 20% reduction the study team aimed to achieve. Nevertheless, the greater reduction in antibiotic prescription rates achieved by Meeker et al may be because they conducted their study in an ambulatory setting and thus physicians were more assured of follow-up and able to develop a rapport with their patients. Furthermore, their posted signed commitment letters were accompanied by a photograph of the physician, which increases identifiability and thus the provider’s obligation to antibiotic stewardship. The electronic signatures used on our signed commitment collectively identified the ED providers without an accompanying photograph, which may have resulted in a diffusion of responsibility and a decrease in motivation to adhere to antibiotic prescription guidelines. Also, the Meeker et al study individually recruited physicians who knew the study team was monitoring their antibiotic prescription rates. Although the ED providers in our study were also aware of the intervention, they knew that the ED AOM antibiotic prescription rates would be viewed collectively and not analyzed by each individual provider.

### Implications

Traditional methods of changing physician behavior involve using educational approaches, financial incentives, audit and feedback in which physicians receive suggestions for improvement on their performance, and systems-based interventions. Multifaceted approaches and active methods (such as workshops and outreach programs) are more effective at altering behavior than passive education methods.^18^ A Cochrane Review that evaluated financial incentives showed that they improved certain areas of care (admissions/referrals and care processes such as healthcare utilization/costs), but did not affect compliance with guidelines.^19^ A recent systematic review evaluating interventions aimed at reducing imaging and laboratory tests found that the most effective interventions utilized education that targeted both families and clinicians, audit and feedback, and systems-based interventions (changes in institutional structure and changes in the EMR).^20^

Although not designed with behavioral economics in mind, most traditional methods and interventions use principles from this field, and the interventions’ successes are often framed in this context.^21,22^ Research from behavioral economics suggests that providing information alone is not enough to induce change and that people tend to resist change due to inertia.^21,23^ Moreover, people have difficulty making choices if options are abundant, suggesting that having fewer EMR alerts or focusing on a balance of performance metrics can yield a greater degree of behavioral change.^24^ For motivating behavior, people discount delayed benefits (preferring immediate gratification), respond better to discrete, frequent rewards, and are loss-averse.^25^ Social comparison motivates people to improve their performance to match top-performing peers.^26^ To maintain change, systems-based changes that facilitate targeted behavior should also be implemented to avoid relying solely on a person’s internal motivation for persistent optimal behavior.^27^

Employing these principles can be useful in designing interventions in the future. This study demonstrated increased ED provider adherence to AAP guidelines for AOM management through an educational approach that targeted both the providers and the patients’ families and incorporated the principle of public commitment through relying on providers’ accountability to their patients and colleagues in the form of a commitment poster. These interventions produced modifications in provider behavior in the short term. The simplicity and generalizability of these interventions render them attractive for application in changing provider behavior in many different settings for a variety of disease processes.

### Limitations

This study had several limitations. It was a retrospective study and thus limited to the data already available and unable to accurately verify AOM diagnoses. Not all providers received all intervention elements. Specifically, not all were present for the 1-hour long presentation on guidelines and the QI project, and we did not formally track who reviewed the reminder email content and how thoroughly. We did not link adherence to guidelines rates with specific providers to know if those who had received all intervention elements were more likely to have improvement in their rates. We relied on provider documentation in the EMR to determine if they educated patients regarding WW. The study authors did not track balancing measures such as failed WW, antibiotic treatment failure, or side effects of prescribed medications. However, in our postintervention cohort, 2 patients who qualified for immediate antibiotics underwent WW instead, suggesting that this balancing measure should be closely monitored to prevent delay in AOM treatment. Most importantly, the study’s timespan was limited to 1 month after the completion of all interventions, and additional research is needed to determine if these interventions produced long-term effects in modifying provider behavior. Last, because we implemented multiple interventions at once, it is difficult to differentiate the discrete effect each intervention had in improving adherence to AAP AOM management guidelines.

## CONCLUSIONS

A multifaceted approach to reduce antibiotic prescription rates for AOM management in a pediatric ED improved adherence to AAP guidelines for WW by reducing immediate antibiotic prescriptions while concurrently increasing WW. These findings show that interventions designed to educate providers and families, expedite family education, and appeal to providers’ commitment to their patients while holding them responsible to guideline-based care in the eyes of their peers, can modify provider behavior in the short term.

## ACKNOWLEDGMENTS

We would like to thank Phung K. Pham, MS, MA, for her assistance with an audit of the study methodology and statistical analyses.

## DISCLOSURE

The authors have no financial interest to declare in relation to the content of this article.

## Supplementary Material
