# Supplementary figures and images for: A Multifaceted Quality Improvement Intervention to Improve Watchful Waiting in Acute Otitis Media Management
Source: Pediatr Qual Saf. 2019 May 23;4(3):e177. doi: 10.1097/pq9.0000000000000177 (PMC6594788; doi:10.1097/pq9.0000000000000177)

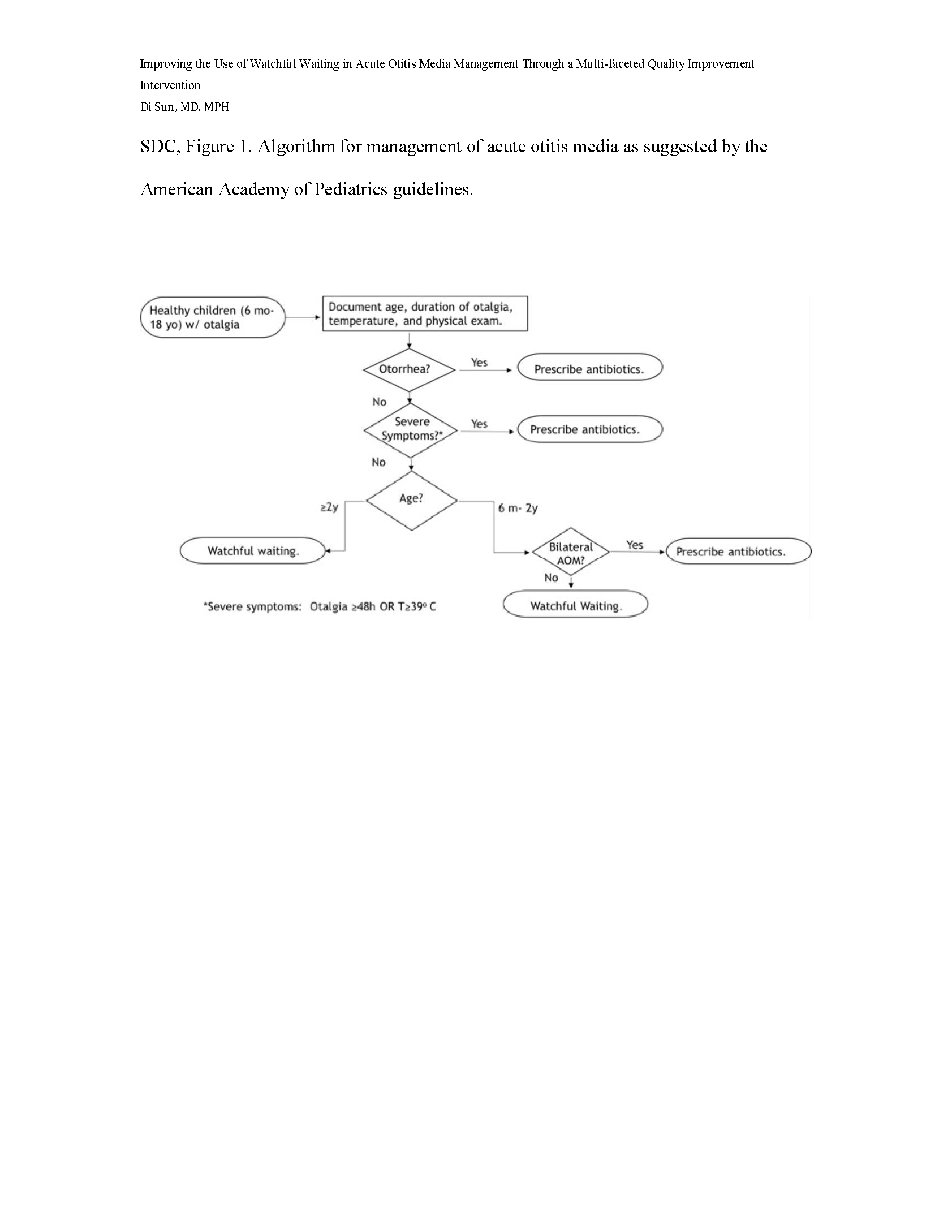

Supplement: Supplementary file 1 [file pqs-4-e177-s001.tif]

SDC, Figure 2. Acute otitis media handout explaining watchful waiting for families.


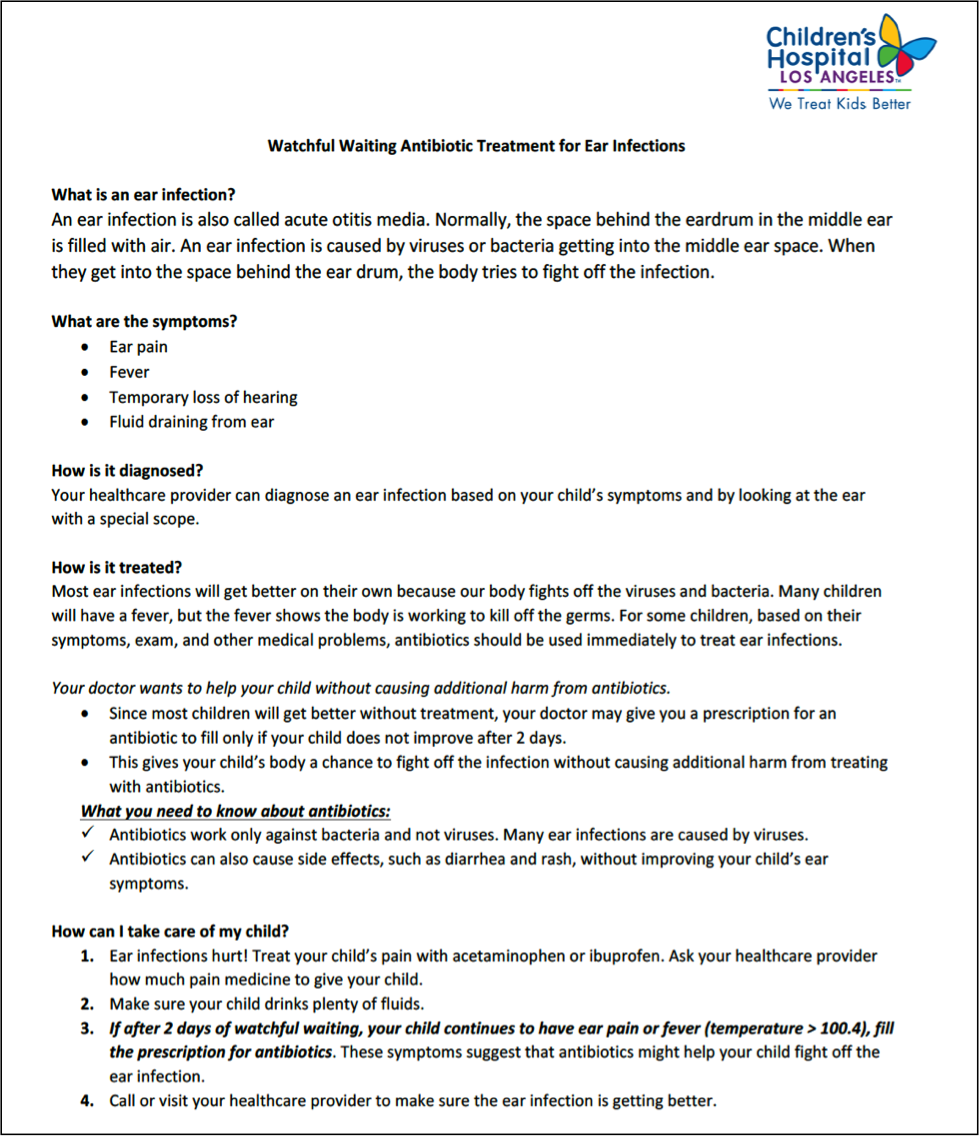

Supplement: Supplementary file 2 [file pqs-4-e177-s002.docx]

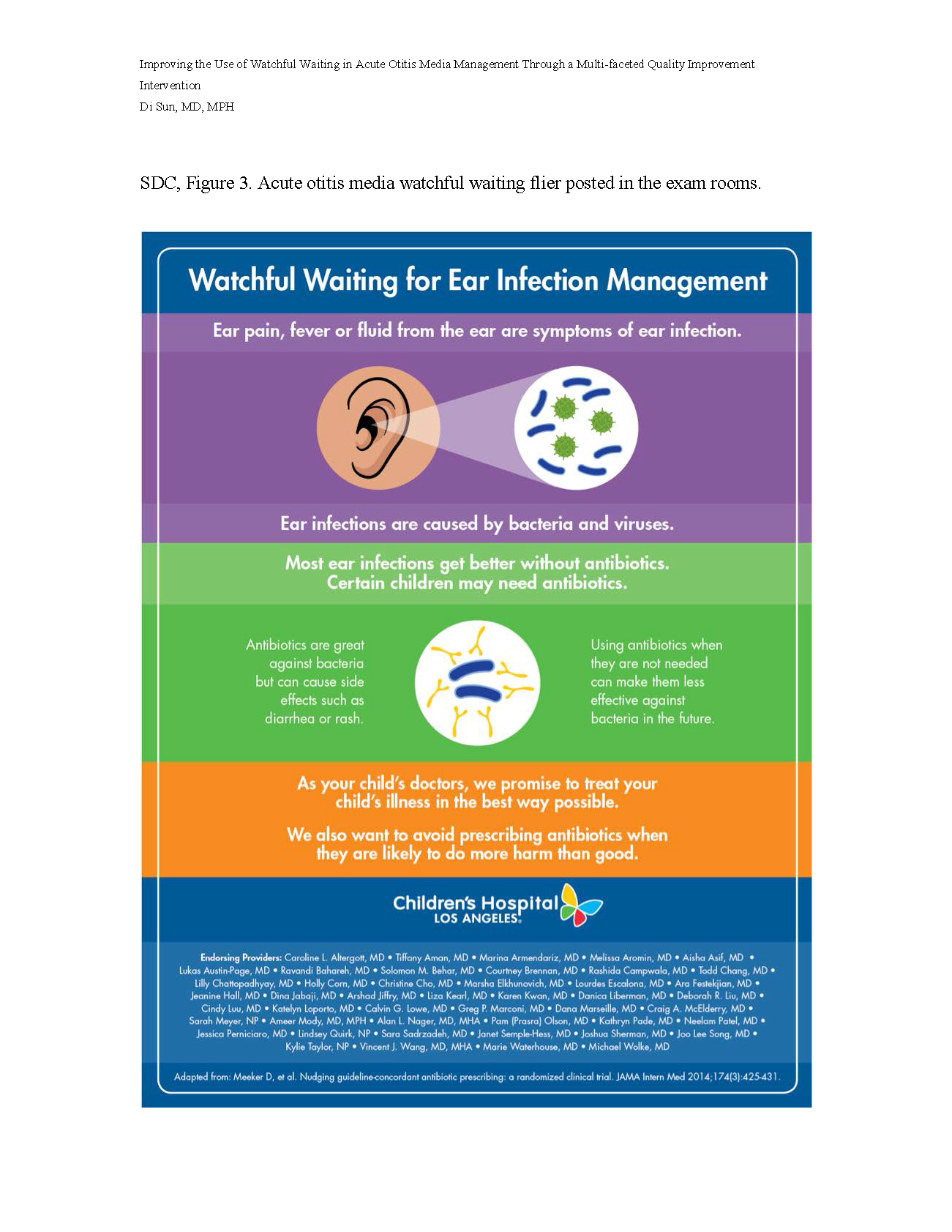

Supplement: Supplementary file 3 [file pqs-4-e177-s003.tif]
